# Impact of Antifungal Prophylaxis Continuation or Discontinuation After Allogeneic Hematopoietic Cell Transplant on the Incidence of Invasive Mold Infection

**DOI:** 10.1093/ofid/ofae409

**Published:** 2024-07-23

**Authors:** Justine Abella Ross, Brian Lee, Huiyan Ma, Bernard Tegtmeier, Deepa Nanayakkara, Jana Dickter, Ricardo Spielberger, Eileen Smith, Vinod Pullarkat, Stephen J Forman, Randy Taplitz, Ryotaro Nakamura, Monzr Al Malki, Sanjeet Singh Dadwal

**Affiliations:** Department of Pharmacy, City of Hope National Medical Center, Duarte, California, USA; Department of Pharmacy, City of Hope National Medical Center, Duarte, California, USA; Division of Biostatistics, Department of Computational and Quantitative Medicine, Beckman Research Institute of City of Hope, Duarte, California, USA; Department of Quality Risk and Regulatory Management, City of Hope National Medical Center, Duarte, California, USA; Division of Infectious Disease, City of Hope National Medical Center, Duarte, California, USA; Division of Infectious Disease, City of Hope National Medical Center, Duarte, California, USA; Department of Hematology and Hematopoietic Cell Transplantation, City of Hope National Medical Center, Duarte, California, USA; Department of Hematology and Hematopoietic Cell Transplantation, City of Hope National Medical Center, Duarte, California, USA; Department of Hematology and Hematopoietic Cell Transplantation, City of Hope National Medical Center, Duarte, California, USA; Department of Hematology and Hematopoietic Cell Transplantation, City of Hope National Medical Center, Duarte, California, USA; Department of Medicine, City of Hope National Medical Center, Duarte, California, USA; Department of Hematology and Hematopoietic Cell Transplantation, City of Hope National Medical Center, Duarte, California, USA; Department of Hematology and Hematopoietic Cell Transplantation, City of Hope National Medical Center, Duarte, California, USA; Division of Infectious Disease, City of Hope National Medical Center, Duarte, California, USA

**Keywords:** antifungal, hematopoietic, invasive mold infection, prophylaxis, transplant

## Abstract

**Background:**

Continuing antifungal prophylaxis (AFPx) to prevent invasive mold infections (IMIs) in recipients of allogeneic hematopoietic cell transplantation (alloHCT) after primary hospital discharge from alloHCT admission varies among transplant centers despite recommendations to continue prophylaxis through day +75. Characteristics driving AFPx prescribing at hospital discharge and outcomes are unknown.

**Methods:**

In this retrospective analysis, we reviewed patients continuing AFPx vs no AFPx at hospital discharge. We included patients with a hospital stay ≥7 days and ≤40 days. We excluded patients with a history of IMI prior to alloHCT, new IMI during admission, or death prior to discharge. Our primary objective was incidence of probable or proven IMI per the European Organization for Research and Treatment of Cancer and the Mycoses Study Group Education and Research Consortium. Our secondary objectives were nonrelapse mortality at day +100, overall survival at day +100, and characteristics driving AFPx discontinuation at hospital discharge.

**Results:**

Of the 430 patients identified, 387 met inclusion criteria. At discharge, 56% (217/387) continued AFPx, and 44% (170/387) had no AFPx. At day +100, 3 probable IMI cases occurred in the group with continued AFPx vs 1 probable IMI case in the no-AFPx group (no proven IMI). Univariate analysis showed no difference in cumulative incidence of probable IMI (*P* = .440), nonrelapse mortality (*P* = .072), and overall survival (*P* = .855) between groups. Multivariable logistic regression demonstrated that patients were less likely to continue AFPx if they had a diagnosis other than acute myeloid leukemia, a length of stay ≤30 days, acute graft-vs-host disease grade 0 or 1, and corticosteroid use ≤5 days.

**Conclusions:**

There was no difference in probable IMI at day +100 after alloHCT based on continuing vs discontinuing AFPx at hospital discharge after alloHCT admission supporting a risk-adapted prophylaxis approach.

## Teaser

Patients with a diagnosis other than acute myelogenous leukemia, a length of stay ≤30 days, a maximum grade <2 for graft-vs-host disease, and ≤5 days of corticosteroids are less likely to continue antifungal prophylaxis upon discharge after primary allogeneic hematopoietic cell transplantation.

Invasive mold infection (IMI) is a serious complication after allogeneic hematopoietic cell transplantation (alloHCT). In 1995, a randomized clinical trial in patients who received fluconazole prophylaxis in the first 75 days after alloHCT exhibited a survival benefit, despite its lack of antimold activity [1]. In 2004, a study comparing micafungin vs fluconazole showed that micafungin was successful in preventing *Candida* spp and *Aspergillus* spp during the neutropenic phase after alloHCT [2]. There has been an epidemiologic shift over the last few decades with the majority of IMI now caused by *Aspergillus* spp or other molds leading to poor survival outcomes [3, 4]. Risk factors for IMI, particularly *Aspergillus* spp, in the first 100 days after alloHCT are driven by (1) host factors (eg, donor type, stem cell source, age, prior environmental exposure, iron overload status), (2) transplant-related variables (conditioning regimen–myeloablative vs nonmyeloablative, use of posttransplant cyclophosphamide, donor match level), and (3) clinical complications (eg, acute graft-vs-host disease [GVHD], which involves varying levels of immunosuppression; cytomegalovirus infection) [5]. The degree and duration of neutropenia and lymphopenia also play a role in risk for IMI during all periods post-alloHCT [4]. Although guideline recommended, universal antifungal prophylaxis through day +75 post-alloHCT introduces the possibility of developing antifungal resistance, toxicities from drug-drug interactions and the drug itself, and increased cost [6–8]. Analysis of individual risk factors during the post-alloHCT course by a more refined approach to determine the duration of antifungal prophylaxis in this population should be considered. Risk-stratified prophylactic strategies have been reported in retrospective studies identifying those who may benefit from antifungal prophylaxis, such as those with multiple transplants, but more studies exploring this approach are needed [9]. Recent advancements in targeted chemotherapies and immunotherapies may decrease the risk of IMI, which may warrant an evaluation of the duration of antifungal prophylaxis in this population [10]. Our institution has generally supported the use of antifungal prophylaxis, preferably with posaconazole in allogeneic recipients with GVHD, upon hospital discharge from primary alloHCT admission until day +75 or longer in those with continued risk factors for IMI. Our institution also supports the use of voriconazole as an alternative if insurance barriers preclude the use of posaconazole. In some circumstances, isavuconazole may be used for antifungal prophylaxis in the context of major drug interactions or QTc prolongation or to provide an oral option in cases where micafungin may not be feasible in the ambulatory setting. After primary alloHCT discharge, our HCT program continues to follow patients closely in the ambulatory care setting at a approximate frequency of twice weekly until at least day +100 and once weekly through day +180 (or longer depending on individualized needs). The primary objective of our study is to determine the incidence of probable or proven IMI at day +100 per definitions of the EORTC/MSGERC (European Organization for Research and Treatment of Cancer/Mycoses Study Group Education and Research Consortium), nonrelapse mortality, and overall survival in those continuing antifungal prophylaxis vs no antifungal prophylaxis at primary hospital discharge following a hospital admission for alloHCT [11]. Our secondary objective is to assess the associations of patient characteristics and the likelihood of continuing antifungal prophylaxis.

## METHODS

This single-center retrospective review was approved by the City of Hope National Medical Center Institutional Review Board and deemed exempt from need of informed consent. Patients ≥18 years old undergoing alloHCT from 1 December 2018 to 31 December 2019 were retrospectively reviewed. We included patients admitted for first alloHCT with a length of stay ≥7 days and ≤40 days. Forty days was chosen as a cutoff point to capture the risk of IMI in the early postengraftment period consistent with previous models in the literature [12, 13]. Primary hospital discharge was defined as the first discharge from the hospital following an admission for alloHCT after successful engraftment. Patients were categorized into 2 groups: continuing antifungal prophylaxis (antifungal prescribed ±5 days of hospital discharge) and no antifungal prophylaxis (patients receiving inpatient antifungal prophylaxis but not prescribed any within 5 days of hospital discharge). Patients with a length of stay >40 days, a history of IMI prior to alloHCT, development of IMI during primary alloHCT admission, primary graft failure, or death prior to discharge were excluded. Patients were also excluded if they received no antifungal prophylaxis at hospital discharge but started it at any point within day +75. These patients were excluded to account for factors such as GVHD and its treatments, which may confound patient characteristics driving decision making regarding antifungal prophylaxis prescribing at the time of primary hospital discharge.

### Data Collection

Data included demographics, conditioning regimen, stem cell source, donor type and match, indication for alloHCT, time to engraftment, GVHD, and inpatient corticosteroid use (defined as ≥0.5 mg/kg/d of prednisone equivalent). Probable or proven invasive IMI was defined per the EORTC/MSGERC according to microbiology and pathology databases [11].

### Statistical Analyses

SAS version 9.4 (SAS Institute) was used to perform the analyses. Characteristics of patients with continued vs no antifungal prophylaxis are shown in a contingency table (Table 1). Univariate analysis was conducted to assess the differences in post-alloHCT outcomes between patients with continued antifungal prophylaxis and no antifungal prophylaxis, including day +100 probable/proven IMI, nonrelapse mortality, and overall survival. Day +100 probable/proven IMI and nonrelapse mortality were assessed by cumulative incidence curves and Gray tests, where death was viewed as a competing risk for IMI and relapse was viewed as a competing risk for nonrelapse mortality. Moreover, 1 patient was excluded in the analysis for nonrelapse mortality since it occurred prior to discharge. Overall survival for patients with continued antifungal prophylaxis vs no antifungal prophylaxis were examined with Kaplan-Meier curves and a log-rank test.

**Table 1.** Patient Demographics

	No. (%) or Median (IQR)		
Characteristic	C-AFPx (n = 217)	N-AFPx (n = 170)	Total (n = 387)
Recipient sex			
Female	95 (43.8)	86 (50.6)	181 (46.8)
Male	122 (56.2)	84 (49.4)	206 (53.2)
Primary diagnosis			
Acute lymphocytic leukemia	47 (21.7)	38 (22.4)	85 (22)
Acute myeloid leukemia	116 (53.5)	67 (39.4)	183 (47.3)
Chronic myeloid leukemia	7 (3.2)	4 (2.4)	11 (2.8)
Chronic myelomonocytic leukemia	7 (3.2)	3 (1.8)	10 (2.6)
Myelodysplastic syndrome	19 (8.8)	16 (9.4)	35 (9)
Non-Hodgkin lymphoma	7 (3.2)	14 (8.2)	21 (5.4)
Others	14 (6.5)	28 (16.5)	42 (10.9)
Age at transplant, y	56 (1–76)	57 (19–76)	56 (1–76)
Stem cell source			
Bone marrow	11 (5.1)	10 (5.9)	21 (5.4)
Cord blood	1 (0.5)	0 (0)	1 (0.3)
Peripheral stem cells	205 (94.5)	160 (94.1)	365 (94.3)
Length of stay, d	31 (20–39)	27 (16–39)	29 (16–39)
>30 d of stay			
No	92 (42.4)	130 (76.5)	222 (57.4)
Yes	125 (57.6)	40 (23.5)	165 (42.6)
Time to engraftment, d	16 (10–28)	16 (8–25)	16 (8–28)
Days from transplant to discharge	24 (16–36)	21 (11–33)	23 (11–36)
Conditioning type			
Ablative	102 (47)	69 (40.6)	171 (44.2)
Nonmyeloablative/reduced intensity	115 (53)	101 (59.4)	216 (55.8)
Type of transplant			
Cord	1 (0.5)	0 (0)	1 (0.3)
Haploidentical	31 (14.3)	15 (8.8)	46 (11.9)
MRD	65 (30)	66 (38.8)	131 (33.9)
MUD/MMUD	120 (55.3)	89 (52.4)	209 (54)
GVHD prophylaxis			
Cyclophosphamide based	71 (32.7)	68 (40)	139 (35.9)
Tacrolimus based	144 (66.4)	101 (59.4)	245 (63.3)
Others	2 (0.9)	1 (0.6)	3 (0.8)
Acute GVHD from transplant to discharge			
No	131 (60.4)	154 (90.6)	285 (73.6)
Yes	86 (39.6)	16 (9.4)	102 (26.4)
Maximum grade			
0–1	147 (67.7)	163 (95.9)	310 (80.1)
2	55 (25.3)	6 (3.5)	61 (15.8)
3–4	15 (6.9)	1 (0.6)	16 (4.1)
Received >5 d of high-dose steroids			
No	129 (59.4)	155 (91.2)	284 (73.4)
Yes	88 (40.6)	15 (8.8)	103 (26.6)
Choice of antifungal medication at discharge			
Posaconazole	154 (71)	0 (0)	…
Isavuconazonium sulfate	32 (14.7)	0 (0)	…
Micafungin	16 (7.4)	0 (0)	…
Voriconazole	12 (5.5)	0 (0)	…
Fluconazole	3 (1.4)	0 (0)	…

Abbreviations: C-AFPx, continued antifungal prophylaxis; GVHD, graft-vs-host disease; N-AFPx, no antifungal prophylaxis; MMUD, mismatched unrelated donor; MRD, matched related donor; MUD, matched unrelated donor.

Logistic regression models were used in multivariable analyses to estimate the odds ratios (ORs) and corresponding 95% CIs for the associations of continued antifungal prophylaxis, with patients’ characteristics listed in the contingency table (Table 2) (not including the choice of antifungal medication at discharge). The stepwise backward selection procedure was used, with variables having a univariable *P* < .05 being considered first by keeping variables having a multivariable *P* < .05. Forward selection was then applied for all variables that were included in univariable analysis but not selected into the backward selection model. To avoid the sparse number involved in multivariable analyses, some detailed variables were collapsed into less detailed variables—for example, a 7-category variable for primary diagnosis was combined into a 4-category variable (acute myeloid leukemia [AML], acute lymphocytic leukemia, myelodysplastic syndrome, and others), and a 3-category variable for maximum-grade GVHD was combined into 2-category variable (0–1 vs ≥2). Also, when a 4-category variable for type of transplant was involved in modeling, the category for cord transplant was excluded since only 1 patient was a recipient of cord stem cells. The final multivariable logistic regression model was constructed by keeping variables having a multivariable *P* < .10. The significance of the OR (2-sided *P* value) was assessed by the Wald test.

**Table 2.** Adjusted Odds Ratios and 95% CIs for the Associations of Continued Antifungal Prophylaxis With Patient Characteristics

	No.		Multivariate Analysis	
	C-AFPx	N-AFPx	Adjusted OR^a^ (95% CI)	*P* Value^b^
Recipient sex				
Female	95	86	1 [Reference]	
Male	122	84	1.56 (.96–2.53)	.072
Primary diagnosis				
Acute lymphocytic leukemia	47	38	1 [Reference]	
Acute myeloid leukemia	116	67	1.92 (1.02–3.61)	**.045**
Myelodysplastic syndrome	19	16	0.68 (.26–1.81)	.440
Others	35	49	0.52 (.25–1.09)	.083
>30 d length of stay				
No	92	130	1 [Reference]	
Yes	125	40	3.22 (1.93–5.39)	**<.0001**
Maximum grade of GVHD onset from transplant to discharge				
0–1	147	163	1 [Reference]	
2–4	70	7	5.69 (2.33–13.89)	**.0001**
>5 d of corticosteroids				
No	129	155	1 [Reference]	
Yes	88	15	5.21 (2.69–10.11)	**<.001**

Abbreviations: GVHD, graft-vs-host disease; C-AFPx, continued antifungal prophylaxis; N-AFPx, no antifungal prophylaxis; OR, odds ratio.

^a^ORs (95% CIs) are from multivariate logistic regression model. Characteristics listed in the table are mutually adjusted.

^b^Based on Wald test. Bold indicates *P* < .05.

## RESULTS

**Figure 1.**
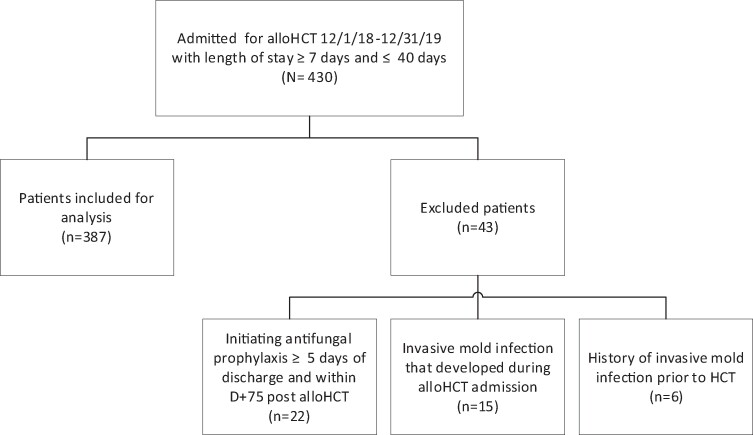
Study design. alloHCT, allogeneic hematopoietic cell transplantation; D, day.

From 1 December 2018 to 31 December 2019, 430 patients admitted for alloHCT had a length of stay ≥7 and ≤40 days. In total 387 patients met inclusion criteria for analysis. Patients were excluded for the following reasons: history of IMI prior to alloHCT (n = 6), IMI that developed during the alloHCT admission (n = 15), and no prescribed antifungal prophylaxis at discharge but initiation of it within day +75 (n = 22; Figure 1). At hospital discharge, 56% (217/387) of patients continued antifungal prophylaxis, and 44% (170/387) had no antifungal prophylaxis prescribed. Table 1 shows patient demographic and baseline characteristics overall and stratified into groups by continued antifungal prophylaxis vs none. Demographic details include age, gender, primary diagnosis, stem cell source, hospital length of stay, conditioning regimens, type of alloHCT, GVHD prophylaxis, and antifungal prescribed at hospital discharge.

**Figure 2.**
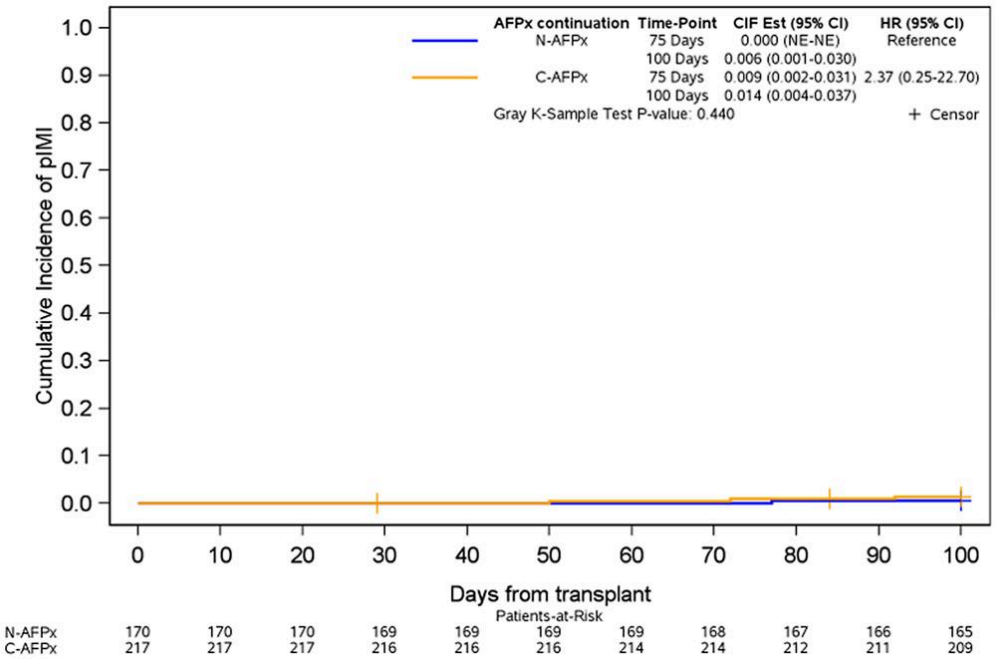
Cumulative incidence of probable invasive mold infections in patients continuing antifungal prophylaxis or not. C-AFPx, continued antifungal prophylaxis; CIF, cumulative incidence function; HR, hazard ratio; N-AFPx, no antifungal prophylaxis; NE, not estimable; pIMI, probable invasive mold infection.

Four IMI events were identified during the first 100 days of alloHCT: 3 of 217 in the continued antifungal prophylaxis group and 1 of 170 in the no-prophylaxis group. Univariate analysis showed no statistically significant difference in day +100 cumulative incidence of IMI between continued antifungal prophylaxis and no antifungal prophylaxis (Gray test, *P* = .440; Figure 2), with day +100 point estimates of 1.4% (95% CI, .4%–3.7%) and 0.6% (95% CI, .1%–3.0%), respectively. All patients with IMI had bronchoalveolar lavage fluid aspergillus galactomannan indexes between 1.18 and 5.46 and none with proven IMI.

**Figure 3.**
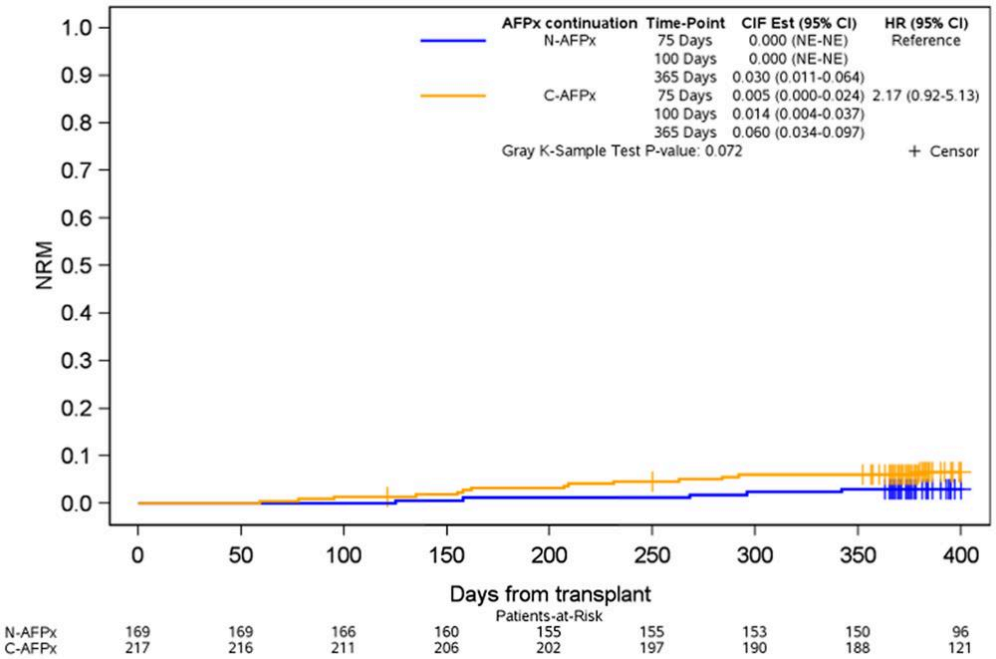
Nonrelapsed mortality for patients continuing antifungal prophylaxis or not. C-AFPx, continued antifungal prophylaxis; CIF, cumulative incidence function; HR, hazard ratio; N-AFPx, no antifungal prophylaxis; NE, not estimable; NRM, nonrelapsed mortality.

Comparison of nonrelapse mortality between the groups in univariate analysis did not show any statistically significant difference (Gray test, *P* = .072; Figure 3). In the continued antifungal prophylaxis group, the nonrelapse mortality point estimates were 1.4% and 6% for day +100 and day +365, respectively. In the group without antifungal prophylaxis, point estimates were 0% and 3% for day +100 and day +365.

**Figure 4.**
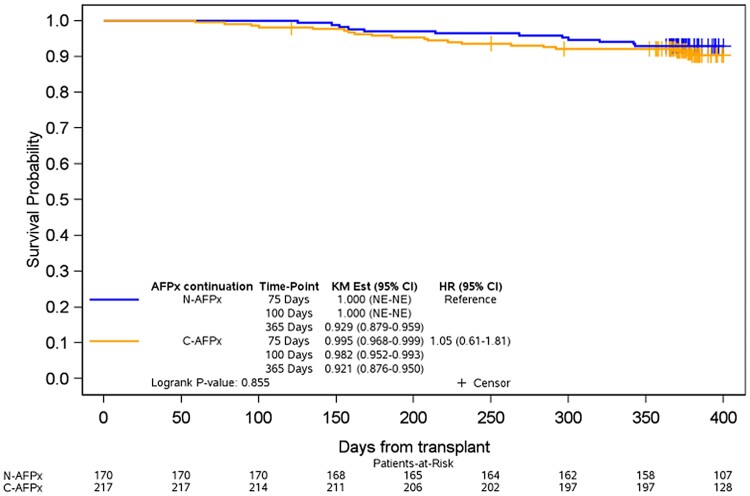
Overall survival for patients continuing antifungal prophylaxis or not. C-AFPx, continued antifungal prophylaxis; HR, hazard ratio; KM, Kaplan-Meier; N-AFPx, no antifungal prophylaxis; NE, not estimable.

Overall survival of patients with continued antifungal prophylaxis was not statistically different from that of patients with no antifungal prophylaxis in univariate analysis (log-rank test, *P* = .855; Figure 4). In the continued antifungal prophylaxis group, the point estimates of overall survival were 98.2% and 92.1% for day +100 and day +365, respectively. In the no-prophylaxis group, point estimates were 100% and 92.9% for day +100 and day +365.

Table 2 shows univariable and multivariable statistical analysis results for the associations between patient characteristics and continuing antifungal prophylaxis after hospital discharge. Multivariable analysis showed that continuing antifungal prophylaxis was less likely to occur in patients as follows:

- With a diagnosis in categories other than AML (vs AML: adjusted OR, 0.52 [95% CI, 0.28–0.98; *P* = .045] for acute lymphocytic leukemia; 0.36 [95% CI, .15–.87; *P* = .023] for myelodysplastic syndrome; 0.27 [95% CI, .14–.50; *P* < .0001] for others)
- With a length of stay ≤30 days vs >30 days (adjusted OR, 0.31; 95% CI, .19–.52, *P* < .0001)
- Without GVHD or with maximum grade 1 GVHD vs maximum grade 2 to 4 (adjusted OR, 0.18; 95% CI, .07–.43; *P* = .0001)
- With a duration of corticosteroids ≤5 days vs >5 days during inpatient admission (adjusted OR, 0.19; 95% CI, .10–.37; *P* < .0001)

Female patients were also less likely to continue antifungal prophylaxis after discharge, but this association did not reach statistical significance (adjusted OR, 0.64; 95% CI, .40–1.04; *P* = .072).

## DISCUSSION

Previous studies have shown low adherence to antifungal guidelines (with antimold active agent) mainly in the inpatient setting with compliance rates ranging from 25% to 75% [14]. Our study is the first to characterize real-world decision making on prescribing antifungal prophylaxis at hospital discharge following admission for alloHCT. In our cohort, 44% of patients were discharged without antifungal prophylaxis despite guideline recommendations to continue it through day +75 (a study that was performed with fluconazole and not an antimold prophylaxis), suggesting incomplete adherence to national guidance [1, 5]. The variability in antifungal prophylaxis prescribing at hospital discharge raised our interest in determining individualized risk factors for IMI rather than strictly following the published guidelines. National guidelines clearly indicate that adoption of the recommendations is voluntary and should not supplant the determination made for each patient by the clinician [15]. In this study, an overall cumulative incidence of IMI was quite low at day +100 at our center (continued vs no antifungal prophylaxis, 1.4% vs 0.6%), which is consistent with recent published literature [16, 17]. In our study, we identified 3 breakthrough probable IMIs in the continued antifungal prophylaxis group that developed with posaconazole (therapeutic levels not available; all patients were taking posaconazole delayed-release tablets). In these breakthrough cases, all patients had grade 2–4 GVHD and received corticosteroids for >10 days as inpatients during their primary alloHCT admission. Breakthrough IMI may have been associated with extensive comorbidities and significant corticosteroid use and may have occurred in the setting of gastrointestinal GVHD amid concern for suboptimal absorption of posaconazole, although not confirmed by therapeutic drug monitoring. All deaths (n = 4) occurred in the continued antifungal prophylaxis group within day +100, including 1 death within the breakthrough IMI cohort. The patient who died with breakthrough IMI had probable invasive pulmonary aspergillosis (bronchoalveolar lavage fluid aspergillus galactomannan index, 5.46) on day +65 that developed with posaconazole. Conversely, only 1 case of probable invasive pulmonary IMI was identified in the no-prophylaxis group, in a matched sibling donor who did not receive antifungal prophylaxis upon primary hospital discharge through day +75. This patient was diagnosed with grade 2 GVHD at day +76, concurrent with the diagnosis of IMI, and was successfully treated with isavuconazole. Overall, our low rate of IMI confirms that antifungal prophylaxis remains an effective strategy in patients at high risk for IMI, particularly GVHD managed with corticosteroids [18, 19]. The observed incidence of IMI in the United States is approximately 5.8% for matched related donors, who confer the lowest risk for IMI as compared with any allogeneic donor type [20]. In addition, experts suggest that the absence of GVHD and a low dose/short duration of corticosteroid use confer a low risk for IMI [10]. Prescribing trends identified in this study show that patients were less likely to continue antifungal prophylaxis beyond hospital discharge after primary alloHCT admission if any of the following applied: a diagnosis other than AML, a shorter length of stay (<30 days), minimal GVHD (grade 0–1), and receipt of inpatient corticosteroids <5 days. This finding thus highlights a potential group of identifiable low-risk patients who may benefit from no antifungal prophylaxis at primary alloHCT hospital discharge, as well as the need for close monitoring of IMI risk and GVHD in the ambulatory care setting. To draw attention to this potential low-risk group, we explored indications for initiating antifungal prophylaxis and IMI rates for a subgroup of excluded patients (n = 22) who were not prescribed any antifungal prophylaxis at discharge but who initiated it within day +75. Antifungal prophylaxis was initiated in 86% (19/22) due to escalation of immunosuppressive therapy secondary to onset of GVHD; 9% (2/22) started prophylaxis for oral thrush; and 5% (1/22) initiated fluconazole to increase tacrolimus levels. Antifungals were not prescribed for treatment of IMI in this subgroup. We identified 2 cases of probable pulmonary IMI (no proven cases) occurring within day +100 (day +77 and day +87) where antifungal prophylaxis with posaconazole was initiated 12 and 18 days after hospital discharge, respectively, for GVHD requiring escalation of immunosuppression in both cases. Of note, all 22 patients in this group received <5 days of corticosteroids during their primary alloHCT admission and had an average length of stay of 29 days (range, 25–39). Our data highlight the potential for a risk-stratified approach toward antifungal prophylaxis prescribing at primary hospital discharge after alloHCT admission, particularly in those at low-risk for IMI. However, one potential benefit worth noting is the advantage of stable calcineurin inhibitor levels when continuing an azole due to its CYP 3A4 inhibition effect [21]. One patient in our subgroup reinitiated fluconazole for the purposes of increasing tacrolimus levels.

This study carries limitations of a single-center retrospective analysis. Although we did not find significant differences in IMIs, nonrelapsed mortality, or overall survival at day +100 or day +365 between continuing and discontinuing antifungal prophylaxis at hospital discharge, it is noteworthy that these findings were from a univariate analysis. We are unable to preclude that the associations of these outcomes may exist but are masked by potential confounding factors. We are also unable to perform a multivariable analysis for potential associations due to our small number of IMIs (n = 4) and deaths (n = 4), although there was a trend toward a higher nonrelapse mortality in the continued antifungal prophylaxis group. However, there is a selection bias between groups due to more GVHD and AML in the continued prophylaxis group, which confer a higher risk of nonrelapse mortality and overall survival. A short follow-up time frame of 100 days after alloHCT limited our ability to detect significant differences in outcomes. Due to this limitation, we explored nonrelapsed mortality at day +365 and found no increase in it after hospital discharge between the groups (*P* = .072). Overall our study provides insight on real-world prescribing decision making in a select group of patients who may benefit from stopping antifungal prophylaxis at primary hospital discharge following alloHCT, with close monitoring for evolving risk factors for IMI in the ambulatory care setting. Our findings suggest that the primary diagnosis of hematologic malignancy, length of stay, GVHD grade, and inpatient corticosteroid use are important factors driving antifungal prophylaxis prescribing in those discharged during the early postengraftment period.

## CONCLUSION

IMI after alloHCT hospital discharge at day +100 was rare despite early discontinuation of antifungal prophylaxis, supporting a risk-adapted approach rather than universal prophylaxis and polypharmacy. However, it remains important to continually monitor for evolving IMI risk with respect to GVHD and initiation of antifungal prophylaxis with escalation of immunosuppressive therapy. Refinement of risk assessment in prospective clinical trials is prudent to inform practices in selecting those who may benefit from stopping antifungal prophylaxis at hospital discharge.

## Notes

***Patient consent*.** This study was reviewed and approved by the City of Hope National Medical Center Institutional Review Board and deemed exempt from need of informed consent.
